# De Garengeot’s Hernia Mimicking Acute Coronary Syndrome and Complicated by Acute Iliofemoral Deep Vein Thrombosis: A Case Report

**DOI:** 10.7759/cureus.114804

**Published:** 2026-08-19

**Authors:** Henrique T Zeferino, Valentina S Muller, Ana Carolina L da Silveira, Celso Paganella Júnior

**Affiliations:** 1 General Surgery, Grupo Hospitalar Conceição, Porto Alegre, BRA; 2 School of Medicine, Universidade do Extremo Sul Catarinense, Criciúma, BRA

**Keywords:** acute appendicitis, acute coronary syndrome, case report, deep vein thrombosis, de garengeot’s hernia, femoral hernia

## Abstract

De Garengeot’s hernia (DGH) is a rare condition defined by the presence of the vermiform appendix within a femoral hernia sac. We report the case of a 65-year-old female smoker on hormone replacement therapy (HRT) with a known history of a right groin hernia who presented with epigastric pain, nausea, and vomiting. Due to elevated troponin levels, she was initially admitted to the Coronary Care Unit (CCU) for suspected acute coronary syndrome (ACS) and started on dual antiplatelet therapy and full anticoagulation. Following a four-day diagnostic workup that ruled out ACS, a targeted physical examination and bedside ultrasound identified an incarcerated femoral hernia. A subsequent contrast-enhanced computed tomography (CT) scan confirmed an incarcerated DGH associated with a concurrent, mechanical deep vein thrombosis (DVT) of the right external iliac and common femoral veins. Despite recent antiplatelet and anticoagulant exposure, the patient underwent an urgent appendectomy and an anterior mesh-based femoral hernia repair with an uneventful recovery.

## Introduction

De Garengeot’s hernia is defined as the presence of the appendix within a femoral hernia, a rare condition described mainly in isolated case reports [1]. It is an uncommon finding, occurring in approximately 0.8% to 0.9% of all femoral hernias [2,3]. Because of its rarity, the true incidence of de Garengeot’s hernia is difficult to assess accurately, with only a few hundred cases reported worldwide [4,5].

Acute appendicitis developing within the femoral hernia sac is even rarer, occurring in only 0.08% to 0.13% of cases [2,3]. This clinical entity presents a significant female predominance, accounting for approximately 81.1% to 83% of all documented patients [3], with a reported mean age at diagnosis of 55 years [6].

This report describes a rare presentation of De Garengeot’s hernia initially simulating acute coronary syndrome (ACS), which was further complicated by deep vein thrombosis (DVT), highlighting the diagnostic challenges of this unusual clinical entity.

## Case presentation

A 65-year-old female patient with a history of active smoking, systemic atheromatosis, and hormone replacement therapy (HRT) presented to the emergency department with epigastric pain, nausea, and vomiting. The patient had a known history of a right groin hernia for at least five months and was awaiting elective surgery. Initially, a complication related to the groin hernia was not suspected. Due to elevated troponin levels and her high-risk cardiovascular profile, she was provisionally diagnosed with ACS, admitted to the coronary care unit (CCU), and started on dual antiplatelet therapy and therapeutic anticoagulation. ACS was ultimately ruled out after the normalization of serial troponin levels, a normal echocardiogram, and a negative CT pulmonary angiogram.

**Figure 1:**
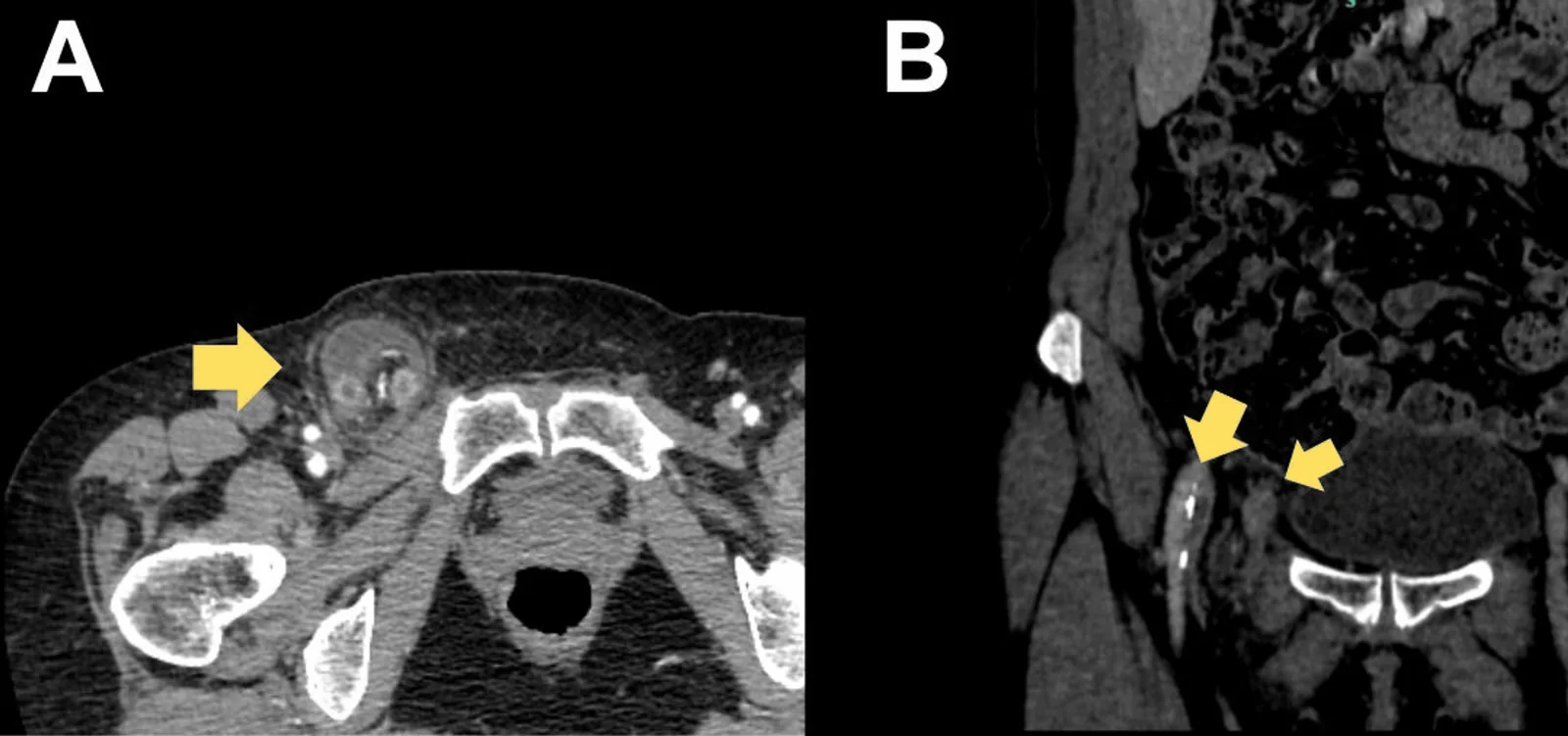
Computed tomography scans showing de Garengeot's hernia and deep vein thrombosis (A) Axial CT view demonstrating the right femoral hernia containing an inflamed vermiform appendix with parietal contrast enhancement and surrounding fat stranding; (B) Coronal CT view demonstrating an acute, extensive deep vein thrombosis (DVT) with a clear intraluminal filling defect involving the right external iliac and common femoral veins (lateral arrow), directly caused by mechanical extrinsic compression from the tense, adjacent hernia sac (medial arrow).

On the fourth day of admission, the patient reported progressive pain adjacent to her known groin mass. Physical examination and abdominal ultrasound revealed an incarcerated femoral hernia containing a tubular structure highly suggestive of the appendix. To complement the investigation and evaluate the undefined local pain, a subsequent contrast-enhanced abdominal and pelvic CT scan was performed. The scan confirmed acute appendicitis within the right femoral hernia sac along with acute DVT involving the right external iliac and common femoral veins, validating the ultrasound findings (Figure 1).

**Figure 2:**
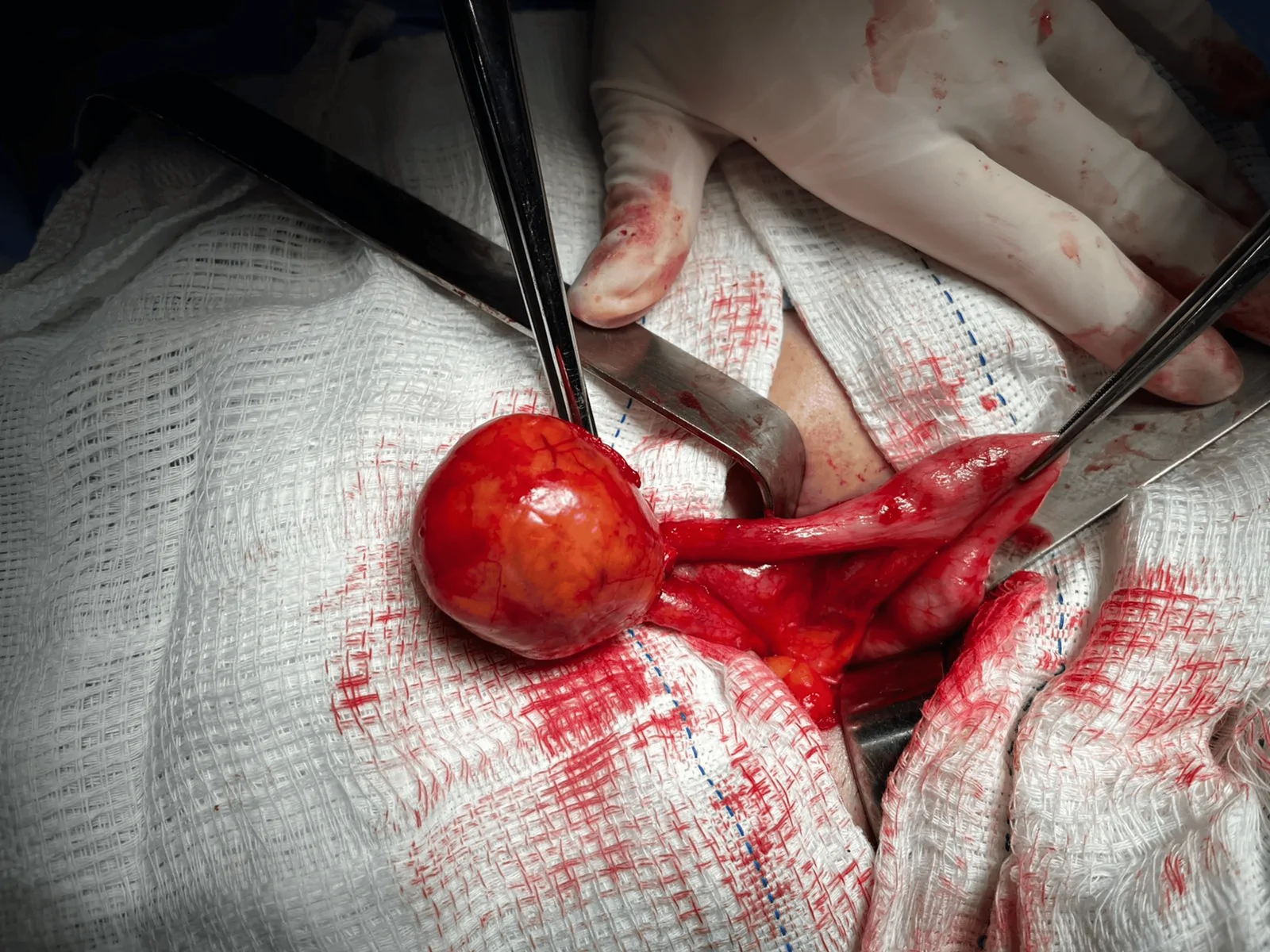
Intraoperative finding of de Garengeot's hernia Intraoperative view of the de Garengeot's hernia showing a thickened, erythematous femoral hernia sac containing the inflamed cecal appendix.

Despite recent exposure to dual antiplatelet therapy and full anticoagulation, urgent surgery was performed via an oblique right inguinal incision, the most commonly reported open approach for the management of De Garengeot's hernia [3,7]. Intraoperatively, a congested and inflamed appendix was identified within the femoral sac (Figure 2).

An appendectomy was performed using base ligation and purse-string inversion.

Following the appendectomy, a prosthetic mesh repair was executed. The inguinal ligament was incised to achieve adequate exposure and safely reduce the hernia contents. A polypropylene mesh was tailored and securely fixed to the pubic tubercle, Gimbernat's (lacunar) ligament, and Cooper's ligament, extending lateral to the femoral vessel margin. The inguinal ligament and the conjoined tendon were then reconstructed using interrupted 2-0 monofilament nylon sutures.

## Discussion

The preoperative diagnosis of DGH remains a significant clinical challenge, established in only 31.5% to 34.7% of cases [3]. Contrast-enhanced CT is the gold standard imaging modality, with a reported sensitivity of 61% to 68%; however, it can often misinterpret the hernia contents as omentum or a lymph node [3,8]. In our patient, CT provided a definitive dual diagnosis by identifying both the femoral hernia and the adjacent vascular thrombosis (Figure 2).

The pathophysiology of appendicitis in DGH is typically attributed to extraluminal compression of the appendix by the narrow, rigid boundaries of the femoral ring, leading to vascular compromise and subsequent bacterial overgrowth [2]. Unlike typical appendicitis, DGH often lacks constitutional symptoms of peritonitis because the narrow femoral canal anatomically isolates the inflammatory process from the free peritoneal cavity [2].

An elegant and treacherous feature of this case was the bilateral mechanical consequence of this rigid ring. While it strangled the appendix medially, the physical expansion and inflammatory edema of the incarcerated sac exerted direct extrinsic compression on the immediately lateral common femoral vein. This extrinsic venous compression fulfills the venous stasis component of Virchow's triad [9], thereby predisposing the patient to acute iliofemoral DVT. Furthermore, the initial elevation of cardiac troponin levels created a major diagnostic dilemma. Given that obstructive coronary disease was ruled out, the transient troponin elevation may be explained by either a Type 2 myocardial infarction, driven by oxygen supply-demand mismatch caused by severe systemic stress, pain, and tachycardia in a patient with underlying systemic atheromatosis [10], or less likely, by subclinical pulmonary microembolization secondary to the acute DVT. 

A critical clinical highlight of this case was the management of emergency surgical hemostasis. The initial misdiagnosis of ACS led to the administration of dual antiplatelet therapy and therapeutic heparin. Performing an urgent appendectomy and an anterior groin hernioplasty under these conditions inherently increases the risk of bleeding, postoperative hematoma, and subsequent surgical site infection. Fortunately, meticulous intraoperative hemostasis and secure anatomical reconstruction prevented these complications, resulting in an uneventful postoperative course with zero hematoma formation.

While the use of prosthetic mesh remains controversial in contaminated surgical fields [11], current consensus in hernia literature supports its deployment in non-perforated cases to effectively reduce the high recurrence rates associated with tissue-only repairs. Based on the classification system proposed by Guenther et al., this case is categorized as Class 2A (erythematous, inflamed, or congested appendix without perforation), which represents the most frequent presentation (44.1%) encountered in clinical practice [12]. In our patient, the deployment of a polypropylene mesh provided a tension-free repair with no infectious complications at the six-month follow-up. Due to the extensive DVT, oral anticoagulation (rivaroxaban) was safely resumed on postoperative day one.

## Conclusions

De Garengeot’s hernia remains a diagnostic challenge that may mimic cardiac or vascular emergencies in elderly patients with complex comorbidities. In this case, early imaging and prompt surgical management allowed successful treatment despite recent dual antiplatelet therapy and anticoagulation. The patient underwent appendectomy and mesh-based femoral hernia repair without bleeding or infectious complications, with an uneventful recovery and safe resumption of anticoagulation. This case underscores the critical role of high clinical suspicion and multidisciplinary coordination in navigating complex, high-risk surgical presentations. Ultimately, personalized perioperative planning is key to optimizing outcomes in multimorbid patients requiring acute intervention.
